# Higher Mosquito Production in Low-Income Neighborhoods of Baltimore and Washington, DC: Understanding Ecological Drivers and Mosquito-Borne Disease Risk in Temperate Cities

**DOI:** 10.3390/ijerph10041505

**Published:** 2013-04-12

**Authors:** Shannon L. LaDeau, Paul T. Leisnham, Dawn Biehler, Danielle Bodner

**Affiliations:** 1 Cary Institute of Ecosystem Studies, Millbrook, NY 12545, USA; 2 Department of Environmental Science and Technology, University of Maryland, College Park, MD 20742, USA; E-Mail: leisnham@umd.edu (P.T.L.); dbodner614@yahoo.com (D.B.); 3 Geography & Environmental Systems, University of Maryland Baltimore County, Baltimore, MD 21250, USA; E-Mail: dbiehler@umbc.edu

**Keywords:** mosquito, urban, vector, *Aedes albopictus*, *Culex pipiens*, income, IPM

## Abstract

Mosquito-vectored pathogens are responsible for devastating human diseases and are (re)emerging in many urban environments. Effective mosquito control in urban landscapes relies on improved understanding of the complex interactions between the ecological and social factors that define where mosquito populations can grow. We compared the density of mosquito habitat and pupae production across economically varying neighborhoods in two temperate U.S. cities (Baltimore, MD and Washington, DC). Seven species of mosquito larvae were recorded. The invasive *Aedes albopictus* was the only species found in all neighborhoods. *Culex pipiens,* a primary vector of West Nile virus (WNV), was most abundant in Baltimore, which also had more tire habitats. Both *Culex* and *Aedes* pupae were more likely to be sampled in neighborhoods categorized as being below median income level in each city and *Aedes* pupae density was also greater in container habitats found in these lower income neighborhoods. We infer that lower income residents may experience greater exposure to potential disease vectors and Baltimore residents specifically, were at greater risk of exposure to the predominant WNV vector. However, we also found that resident-reported mosquito nuisance was not correlated with our measured risk index, indicating a potentially important mismatch between motivation needed to engage participation in control efforts and the relative importance of control among neighborhoods.

## 1. Introduction

Pathogens vectored by arthropods have been a devastating component of global disease burden throughout history [1,2,3] and the most medically important arthropod disease vectors are mosquitoes [4]. Aggressive mosquito control campaigns employed physical and chemical engineering to dramatically reduce human disease burden by the late 1960s [3,5,6], but many regions have experienced a (re)emergence of mosquito-vectored diseases, both due to novel pathogens and those previously eradicated [6,7]. This phenomenon has been particularly evident in the increase in human cases of dengue virus (Family *Flaviviridae,* Genus *Flavivirus*), West Nile virus (WNV, Family *Flaviviridae,* Genus *Flavivirus*), La Crosse virus (LACV, Family *Bunyaviridae*, Genus *Orthobunyavirus*), and chikungunya virus (Family *Togaviridae*, Genus *Alphavirus*) among urban populations across the World [4,8,9,10,11,12,13]. The burden of mosquito-vectored disease is centered in developing regions, but developed nations and modern health care systems have not been spared. In the past decade chikungunya virus has spread into several European cities [14]. La Crosse virus has emerged in the United States (U.S.) and currently spreads with expanding urbanization in the Appalachian region [15]. West Nile virus spread rapidly across the North American continent after its 1999 emergence in New York, although pathogen persistence and endemic transmission are most evident in urban and agricultural landscapes [16,17,18,19,20]. Dengue infections have also increased over the past decade in urban areas along the U.S.-Mexico border [21,22] and in the Florida Keys [23], and spread into other U.S. cities where competent mosquito vectors are already present is a real concern [24].

Human disease incidence of mosquito-vectored pathogens is often directly related to the spatial distribution and abundance of respective vector species [9,20,25]. Despite the long history of pests hitchhiking with humans [26], efforts to examine mosquito infestations in the context of community ecology are relatively recent [27,28,29]. The direct risk to humans from mosquito-vectored disease is undoubtedly influenced by host-vector dynamics at the flighted adult stage [25,30,31,32,33,34], but vector population growth is most directly influenced by ecological processes at the aquatic immature stages. Climate [35], predation [36,37], competition [38,39,40], resource quality and light [40,41] are all important determinants of mosquito larval dynamics and strongly regulate the abundance of biting adults [29,38,42]. There are 176 species of mosquitoes in the United States, ranging from 26 to 85 species in each state [43]. The females of most species require blood meals to produce eggs and many species restrict host-seeking to certain taxonomic groups (e.g., amphibians, birds, or mammals). Thus, there can be strong differences among mosquito species in human-biting behavior and in pathogen infection and transmission efficiencies [25,31,32,33,44,45,46,47,48,49]. Understanding how species composition is determined and when and where abundant vector populations grow are critical components of effective vector management. 

Urbanization seems to facilitate the production of the mosquito species that most often transmit pathogens to humans [2,29]. The most direct effect of urbanization is the creation of habitat (e.g., artificial containers, stormwater pools) that supports the growth and development of immature stages (larva and egg) of some mosquito species [8]. Additionally, urban heat island effects can promote rapid immature development by maintaining high temperature larval habitat [50,51,52,53] and is linked to the introduction and spread of *Aedes* species that preferentially feed on humans [9,54]. Urbanization processes can also act to increase the probability that pathogens are present for transmission. Increased movement of human populations to and between cities and residential expansion into wilderness areas induces ecological shifts in rates of pathogen introduction [15,29,55,56]. Increased human migration to cities from rural areas with endemic pathogens is a common route of urban mosquito-vectored disease outbreaks across developing countries and has been reviewed in past papers [57,58]. Population growth within cities can also result in higher population densities of human blood meals and increased biting and pathogen transmission rates [2,59]. Despite a global research focus, the predictive capacity to identify and effectively manage growing vector populations is still critically limited to broad and course spatio-temporal scales. This is especially true in urban areas where complex and interacting socio-ecological factors ultimately determine mosquito production, species composition, and potential pathogen exposure [60,61,62]. 

In this paper, we begin with a brief review and then present a socio-ecological case study set in two cities in the eastern United States, Baltimore, MD and Washington, DC, to illustrate how understanding the linkages and feedbacks between mosquito ecology and human sociology is critical for effective and sustainable vector control. Our study examines measures of mosquito ecology and human attitudes in five socioeconomically diverse neighborhoods to test the hypothesis that residents in relatively lower income neighborhoods within a city experience greater exposure to human-biting mosquitoes that develop in anthropogenic container habitats. All arthropod vectors are sensitive to environmental changes and urban areas worldwide are experiencing increasingly greater socioeconomic gradients, thus we hope that ideas and approaches in this paper will be relevant beyond understanding mosquitoes in temperate cities in the United States and can inform efforts to understand and manage arthropod disease-vectors in urban areas globally. 

## 2. Mosquitoes and Coupled Natural-Human Systems

Successful and sustainable mosquito control requires both ecological understanding of the pest ecosystem and sociological understanding of the human behaviors and motivations that can either facilitate pest production or support effective control efforts. Mosquito control in an urban landscape is intrinsically linked to political and social forces and built-environment characteristics (e.g., storm water structures, roads, alleys) [60,63,64,65,66]. Socioeconomic status and segregation of economic resources across neighborhoods have a profound effect on ecosystem function, resident engagement, and sustainable revitalization efforts [67,68,69]. Similar socioeconomic forces and disparities are frequently invoked as drivers of mosquito infestations and mosquito-borne disease [70,71,72,73,74,75], but how these play out in developed countries like the U.S. where public health infrastructure is generally viewed as pervasive is of increasing interest [60,61,64,76,77,78]. A 1990 study in California [75] found that automatic watering devices associated with one affluent neighborhood were associated with more breeding habitat and more adult mosquitoes. However, the study found no relationship between measured mosquito abundances (adult or larvae) and resident-reported nuisance activity. This misalignment is particularly problematic given that mosquito control efforts in residential landscapes are often linked to resident complaints. Another California study found that WNV prevalence in vectors and human disease incidence were related to economic conditions and specifically, to location of abandoned swimming pools [61]. Two recent studies that examined *Aedes albopictus* infestations in the northeastern U.S. documented larval presence in 35–68% of water-holding containers, and a majority of individuals sampled were species with potential medical importance [62,78,79,80]. A 2010 study in Washington, DC reported higher densities of *Ae. albopictus* in disused containers relative to other container types and that yards in lower-income neighborhoods had more of these container habitats [61]. A study in New Jersey (NJ) found that discarded tires were an important predictor of *Ae. albopictus* presence in both urban and suburban neighborhoods [61,77]. Dowling *et al.* [76] found that DC residents who reported that they actively emptied or removed container habitats from their yard did have fewer containers with mosquito pupae and that residents in higher socio-economic status neighborhoods had greater knowledge about mosquito ecology but reported less motivation for participating in control activities. Bartlett-Healy [78] implemented a public health education campaign in their NJ neighborhoods and concluded that passive education was not effective at reducing container habitats, although the results did seem to vary across neighborhoods. These studies all highlight the importance of understanding variation in habitat productivity, as well as in resident management and motivation for guiding effective 21st Century mosquito control in urban landscapes. 

Mosquito species that are capable of utilizing anthropogenic water-holding container habitat are of considerable nuisance and medical significance, largely due to their capacity for rapid population growth in habitats where humans are most dense. Ideal habitat is generally shallow, lentic and warm —all characteristics of many anthropogenic container types and some shallow stormwater structures. Larval development and adult population abundances are defined by the persistence and temperature of water in container habitats [53,81,82,83,84,85], and these conditions are influenced by how humans use (or abandon) the containers. An important characteristic of anthropogenic container habitat, whether managed or discarded, is that it can effectively decouple the larval habitat from direct control by more natural environmental processes. For example, tires can hold rainwater long after natural ground pools have dried [86] and water supply to planters is often augmented when rain is sparse. While stormwater management is important in most residential settings and stormwater structures can be sources of adult mosquito production [87,88,89,90], surface water in our focal cities is rapidly routed underground following a storm event. These may still be a potential source of mosquito production in these cities, however since residents lack direct control over these features of the urban environment, we will only mention them tangentially here. 

The history of mosquito control in the United States centers on engineering the built environment and applying chemical treatments to prohibit mosquito breeding and kill adults and these methods have historically been successful at minimizing mosquito and pathogen activity [5], but rely on predictable adult activity and defined larval habitats. For example, programs that use ultra-low volume fogging to kill adults generally apply materials in the evening when air temperature limits evaporation and this is effective for species where adults are active in the evening. Similarly, larvicides (e.g., *Bacillus thuringiensis (Bti)* or methoprene) are effective when applied directly to breeding habitats, but treatment of container habitats is impractical because they are too numerous and often obscure. *Aedes albopictus,* one of the most problematic mosquito species in urban areas globally, is most active during the daytime and matures in small container habitats. Effective control of a species like *Ae. albopictus* requires regular removal or draining of water-holding containers on each property. But public agencies do not have the resources or the legal authority to do this over large areas [91,92]. Thus, sustained and effective mosquito control in residential landscapes increasingly relies on active resident participation.

Diminished funding for municipal services and increasing abundances of container breeding species mean that communities and residents have to accept responsibility to prevent mosquito population growth [93]*.* However, despite assumptions that knowledge of mosquito control is widespread among US residents, few mosquito control programs have successfully empowered communities for effective and sustained control. Many residents are unaware of certain microhabitats in their own yards or do not know how to prevent mosquito proliferation and biting [78,94]. There are parallels to learn from; Baltimore City authorities and Johns Hopkins University researchers made strides in controlling rats during an ambitious housing code-enforcement drive in the 1940s and 50s, but rat populations rebounded within a few years’ time as code violations returned in low-income neighborhoods [95]. Pest control campaigns require maintenance and may not be sustainable if city health authorities fail to engage with residents and leave them with the means to control vectors in the future [95]. 

## 3. Experimental Section

### 3.1. Case Study in Washington, DC and Baltimore, MD

This study examined the sociological and ecological characteristics associated with the distribution and relative abundances of container-utilizing mosquitoes and especially, potential disease vectors across economically diverse neighborhoods in Washington, DC and Baltimore, MD between June and September 2012. Washington and Baltimore metropolitan regions house 5.5 and 2.7 million people, respectively [96]. These cities are located roughly 45 miles apart in the mid-Atlantic region of the United States in a region that boasts some of the highest household income and educational attainment levels in the country. Median household incomes were $63,124 and $38,721 in Washington and Baltimore, respectively in 2011 and 52.5% and 27.5% of residents over 25 years of age have attained a Bachelor’s Degree or higher [96]. However, both Washington and Baltimore also have a high percentage of households subsisting below the poverty line (15.4% and 19.5%) relative to the nationwide rate of 11.1%. These statistics describe two cities with substantial socio-economic variation among neighborhoods. In Washington for example, median household incomes range from $39,302 to $105,366 among culturally distinct neighborhoods. The cities differ in recent population trajectories; while Baltimore’s population declined 3.0% between 2000 and 2006 leaving more than 30,000 vacant properties [97], the Washington population grew by 3.5% in the same time period. Responsibility for the care of vacant lots is often ambiguous, as a large number in the neighborhoods sampled are titled to absentee owners and the city lacks funds to monitor and maintain even publicly-owned lots [97]. The broader Washington-Baltimore Metropolitan region is a hub for immigration, travel and trade and thus, is at a relatively high risk of imported cases of exotic mosquito-vectored diseases [96]. However, like many U.S. cities, Washington and Baltimore have not budgeted for extensive spraying or source reduction programs. In Washington, adult fogging only occurs when a mosquito or human tests positive for WNV [98]. The control area is usually limited to 3–5 blocks and spraying extends for only a few days to provide temporary relief from biting adults. Baltimore City has no consistent mosquito monitoring or control program. Baltimore and Washington represent many urban communities across the globe that are underserved for mosquito control and lack comprehensive policies and plans to deal with vectors.

### 3.2. Mosquito Species in Northeastern United States

Some of the most commonly sampled species in our focal region such as *Aedes albopictus*, *Culex pipiens, Ae. triseriatus*, and *Ae. japonicus* are potential vectors of human disease [15,99,100,101,102,103,104,105]. Since its invasion in the mid-1980s from Japan, *Ae. albopictus* has emerged as the most common human biting mosquito in many eastern U.S. cities [54,105]. *Aedes albopictus* larvae can efficiently utilize even small water-holding containers [79,106,107] and this species’ competitive abilities have already led to shifts in resident species abundances and mosquito community composition [29]. The spread of *Ae. albopictus* has resulted in increased nuisance biting and complaints, owing to its aggressive day-time biting behavior and the ineffectiveness of conventional abatement methods [107]. Resident mosquito species can persist in the presence of *Ae. albopictus* [79,108], including the predominant WNV vector, *Culex pipiens* [46,109] and a more recent invader from Asia, *Ae. japonicus*. *Aedes japonicus* invaded North America in the late 1990s via used tires from Japan [110], and has since spread along the mid-Atlantic seaboard [111,112,113]. While *Ae. japonicus* is not known to aggressively bite humans, it is a competent laboratory vector of La Crosse virus [114], West Nile virus [115], eastern equine encephalitis virus [116] and St. Louis encephalitis virus [117]. 

### 3.3. Sampling Protocol

We selected five row house neighborhoods for this project based on relative median (household) income levels in each city. In Washington neighborhoods ranged from 186–253 hectares and were chosen to represent median incomes below (Trinidad), at (Petworth), and above (Georgetown) the city-wide median household income ($63,124 [96]). For logistical reasons, work in Baltimore was restricted to neighborhoods below (Franklin Square) and at (Union Square) city-wide median household income ($38,721 [96]). Population densities in Washington neighborhoods ranged from 32 people per hectare in Trinidad to 43 people per hectare in Petworth. Neighborhoods in Baltimore were also considerably smaller (22–58 hectares) with population densities of 61 people and 65 people per hectare in Union Square and Franklin Square, respectively. We used median-income to order the focal neighborhoods from lower to higher income categories in each city. We assume that relative median household income levels among neighborhoods within a city reflect a gradient in a broad array of social and economic characteristics, including access to city resources to address pest infestations. The five neighborhoods will be referred to as N1 to N5, where N1 and N2 are the Baltimore neighborhoods from below and at median income and N3, N4, and N5 are the below, median, and above median income neighborhoods in Washington (Table 1). Although it is not our intent to extend inference beyond the comparison of city-specific relative income categories, N2 in Baltimore and N3 in Washington have similar dollar value median household income around $40,000. Each of these neighborhoods are predominantly comprised of row houses. Only households that were row houses and were consistent in structure that defined the neighborhood were selected.

Each neighborhood was visited three times between June 15 and September 15, 2012. An initial list of addresses was generated for each visit. In Washington, the initial parcels (households) were located at least two city blocks away from each other. If residents were not home at a selected parcel, we continued to approach adjacent homes until permission to sample was granted. The process was repeated with additional houses in each of the three visits, with the goal of sampling as many parcels per neighborhood as possible. In Baltimore, where the neighborhoods were considerably smaller in area, sampling was focused on three randomly selected city blocks within each neighborhood with the goal of sampling as many parcels per block in each neighborhood as possible over the three visits. In Baltimore many of the individual parcels open up into a common back alley or green space and sampling effort in Baltimore did not rely as directly on resident-facilitated access to each back yard as it did in Washington. In Baltimore we were able to sample more parcels than in Washington, although not every parcel sampled was matched with a resident questionnaire. 

A total of 94 individual parcels were used for this analysis (mean = 19 per neighborhood). At each occupied parcel, researchers conducted a brief survey about resident (adult only) experience with mosquitoes. For this case study we evaluated only the resident response to the question: “How often are you bothered by mosquitoes?”, which had multiple answer choices: “never”, “monthly”, “weekly”, and “every day”. Researchers sampled up to 1 liter of homogenized water from all accessible water-holding containers. Container water volume and description were recorded. Although tree holes and other vegetation pools were searched for, none were found to be water-holding during our sampling efforts. For analyses, we categorized each container description by its relevant purpose: storage, yard care, recreation, structural and trash. Storage included anything that was clearly meant for storage (e.g., lidded bins, coolers). Yard containers included birdbaths, buckets, planters, watering cans, and garbage cans. Recreation container habitats were small children's pools, sandboxes, sporting equipment and toys. Structural habitats included both ground puddles under air conditioning units or in cracked cement and drainage pipes. Trash included anything that was obviously discarded and not intended for future use (e.g., plastic cups, Styrofoam bowls, plastic bags, cans). Rubber tires were originally categorized as trash, but were also analyzed as a separate category. Sampled mosquito larvae and pupae were returned to lab, enumerated and preserved in ethanol. A representative sample of up to 50 third and fourth instar larvae were identified to species and up to 50 first and second instar larvae to genus using an established key [118]. Pupae were identified to genus based on clear diagnostic differences. For each container, we calculated the densities (per L of water) of larvae by species and each genus of pupae. For the purposes of this study, analyses focus primarily on pupae presence and abundance because this developmental stage is our best estimate of adult production from these container habitats. We also evaluate statistical patterns in presence and abundance of late instar larvae identified to species, which provide an indicator of potential adult production and species composition of our sampled pupae. 

### 3.4. Analyses

We summarized total containers and abundance of each container purpose category per parcel using frequency tables. Statistical associations between the frequencies of each container type and neighborhood income classification were evaluated after controlling for sampling date in a generalized linear model (Poisson link). Both mosquito presence and density measures involved data collected at the container level and there were usually multiple containers per parcel. Further, each parcel was associated with a specific neighborhood. We used multi-level generalized linear regressions to accommodate this hierarchical sampling structure using either Poisson (density response) or binomial (presence response) links. The random neighborhood and household (within neighborhood) effects capture the dependence structure implicit in our sampling regime and account for non-independent variation among containers within a single yard and among houses within a neighborhood. We found no significant clustering of households within blocks in our Baltimore samples and did not include block in further analyses. All variables with an effect size associated with a *p*-value < 0.10 in a univariate (plus random effect) model were included in a full model and the final model for each dependent variable was determined by removing those variables that were no longer significant. Statistical results in text are displayed as (Z statistic, *p*-value) and are evaluated at α = 0.05. Analyses were done in the statistical software R using the multi-level regression package lme4 [119]. 

## 4. Results and Discussion

### 4.1. Species Composition

We sampled 198 water holding container habitats, ranging from 19 habitats sampled in N5 to 70 in N1 (mean = 39.6 per neighborhood). There were twenty-seven container habitats found in Baltimore (20 in Franklin Square) that were not clearly associated with an occupied parcel. These included a range of container types, including trash, buckets, bags, and six tires. These samples were included in analyses of container and mosquito abundance measures for the neighborhoods they were located in (and each was associated with closest parcel ID for hierarchical analyses). The volume of water in each of the container habitats ranged from (estimated) 0.01 L to 100 L. Mean volume was greatest in recycling bins (15.20 +/− 21.35), garbage cans (5.95 +/− 16.02) and buckets (6.36 +/− 13.65). Water volume was not significantly associated with density of *Aedes* larvae or *Culex* and *Aedes* pupae. *Culex* larvae were negatively associated with volume (z = −4.545, *p* *=* 5.49e−06). There were no significant differences in container volume among neighborhoods (all pairwise comparisons *p* > 0.100).

Seven species of mosquito larvae were sampled across the two cities. *Aedes albopictus* sampled accounted for 69.82% of all individuals and was the only species found in all five neighborhoods, ranging from 30.6% to 99.8% of individuals per neighborhood. *Culex pipiens*, *Culex restuans*, and *Aedes triseriatus* constituted 98.9% of all remaining individuals sampled (Table 1). *Aedes aegypti* and *Toxorhynchites rutilus* *septentrionalis* were only sampled in one Washington neighborhood (N3) and occurred at low relative abundances (<0.1%). The invasive *Aedes japonicus* was only sampled in the two Baltimore neighborhoods (N1 and N2) and composed less than 0.1% of the individuals in those neighborhoods. *Culex* larvae accounted for 39.6% of samples in Baltimore *versus* 0.60% in DC and 94.8% of all *Culex* pupae sampled were in Baltimore. *Aedes albopictus* larvae sampled were more evenly distributed between the cities (57.5% in Baltimore), although 77.5% of *Aedes* pupae were sampled from Baltimore neighborhoods and more than half were found in the lowest income neighborhood (N1). 

**Table 1:** Neighborhood classification and mosquito (larvae) species composition.

Neighborhood	City	Relative Median Income	*Culex pipiens*	*Culex restuans*	*Aedes albopictus*	*Aedes triseriatus*
N1	Baltimore	L	12.72%	0.29%	83.09%	3.51%
N2	Baltimore	M	53.66%	14.81%	30.64%	0.48%
N3	Washington	L	1.76%	0.08%	93.57%	4.21%
N4	Washington	M	0.16%	0.00%	99.84%	0.00%
N5	Washington	H	0.00%	0.00%	90.27%	9.73%

### 4.2. Potential Vector Production across Relative Income Categories

The two most common species that were sampled are also the two most likely potential disease vectors in our focal region. *Culex pipiens* is the predominant WNV vector in this region [34,48] and *Ae. albopictus* is a competent vector of several pathogens with potential for importation/introduction to the region in coming decades, including dengue and chikungunya [104,105]. *Aedes* and *Culex* pupae densities (per container habitat) were not significantly different among neighborhoods (all pairwise comparisons *p* > 0.100), nor between the two cities (*p* = 0.989 and *p* = 0.633 for *Culex* and *Aedes*, respectively). Variation in densities of sampled pupae among containers within a parcel (variance = 246.630 and 18.292 for *Culex* and *Aedes*, respectively) were over an order of magnitude greater than neighborhood-level variances (9.241 and 0.817, respectively).

**Figure 1:**
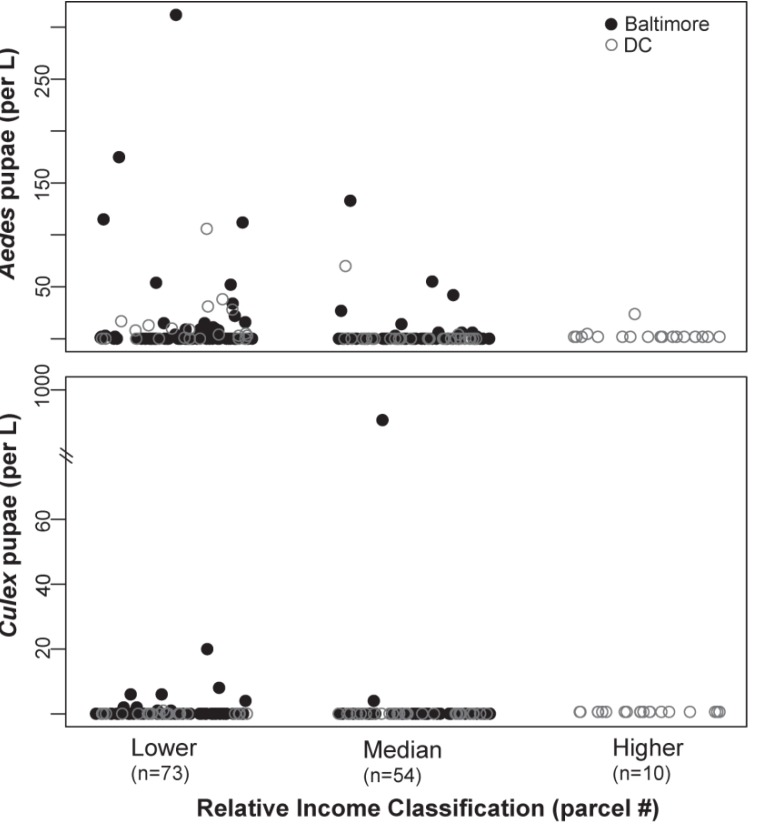
Pupae densities were higher in Baltimore container habitats and in lower income category neighborhoods within each city. Note break in y-axis in bottom panel due to high *Culex* pupae counts in one tire habitat in N2.

*Culex* pupae were more likely to be found in lower income neighborhoods and when present, densities ranged across an order of magnitude (Figure 1). *Culex* pupae were up to 51.80% less likely to be present with each increase in income classification (z = −2.319, *p* = 0.020). However, while relative abundances per container did increase over the season (z = 37.120, *p* < 0.001), density of *Culex* pupae was not associated with neighborhood income classification (Figure 1, z = −0.095, *p* = 0.924). The apparent outlier in the bottom panel of Figure 1 depicts high *Culex* pupae density in a sampled tire habitat in N2 (see Section 4.3). Repeating the above analyses without this extreme habitat does not change the statistical significance of the results. *Culex pipiens* larvae were also sampled at higher densities later in the season (z = 26.790, *p* < 0.001). 

*Aedes* pupae were up to 36.22% less likely to be found with each increase in income classification (z = −3.110, *p* = 0.002) and more likely to be found later in the season (1.972, *p* = 0.049). The density of *Aedes* pupae per container also increased later in the season (z = 6.052, *p* < 0.001) and fewer pupae were found in containers in higher income neighborhoods (Figure 1, z = −2.77, *p* = 0.023). These patterns were consistent for *Ae. albopictus* larvae, which were found at higher densities later in the season (z = 13.802, *p* < 0.001) and at lower densities in higher income neighborhoods (Figure 1; z = 3.177, *p* = 0.001).

### 4.3. The Composition of Container Habitats

There were more water holding containers per parcel sampled in Washington (2.634 +/− 2.318) relative to Baltimore (1.884 +/− 1.351), although this was not statistically significant when clustering within neighborhoods was included (z = 1.721, *p* = 0.085). Abundances of water-holding containers classified as structural (z = 2.379, *p* = 0.017) and storage (z = 2.772, *p* = 0.006) increased during the season, although sample week was not a significant predictor of any other container type. Income classification was not a significant predictor of total numbers of water-holding container habitats per parcel (z = 1.117, *p* = 0.267). Mean abundances per parcel for each income classification and container purpose category are displayed in Table 2. There were significantly more recreation (z = 2.677, *p* = 0.007) and storage (z = 2.938, *p* = 0.003) containers in Washington parcels relative to Baltimore and no differences among numbers of structural or yard containers. Parcels in Washington did tend to have fewer trash containers than were sampled in Baltimore (z = −1.830, *p* = 0.067). In addition to having more trash containers overall, all tire samples were taken from Baltimore neighborhoods. Because both presence and abundance of pupae were generally high in tires, we separated these habitats from the broader trash category for the remainder of the analyses. 

**Table 2:** Container composition and statistical results. Regression coefficients (mean with standard deviation) and p-value shown only if significantly different from 0.

Container Function	Container # per parcel			Density Coefficient (pupae)	
	Lower	Median	Higher	*Culex*	*Aedes*
Storage	0.133	0.087	0.222	ns	2.19 (0.17) *p* < 0.001
Recreation	0.067	0.065	0.111	ns	ns
Structural	0.100	0.087	0.444	ns	ns
Yard care	0.666	1.152	1.222	ns	1.53 (0.13) *p* < 0.001
Trash	0.567	0.500	0.000	ns	ns
Tires	0.375	0.057	0.000	5.83 (1.95) *p* = 0.003	3.14 (0.53) *p* < 0.001

Density of *Aedes* pupae was greater in storage, yard, and tire habitats relative to other habitat types (Table 2). The most common yard purposed container habitats that contained *Aedes* pupae were planters (36.59% had pupae) and buckets (29.10% had pupae). The most common storage containers with *Aedes* pupae were tarps (52.94% had pupae). Tires were the only container habitat type that was a significant predictor of either *Culex* or *Aedes* pupae presence. *Culex* pupae were 36.4% more likely to be found in tires relative to all other container categories (z = 2.141, *p* = 0.032) and *Aedes* pupae were 53.5% more likely to be sampled from tires (z = 2.525, *p* = 0.010). Both *Culex* and *Aedes* were also sampled at higher densities from tire habitats (Table 2). All (17) tires sampled were located in Baltimore and fifteen were in the lowest income neighborhood (N1). *Culex* pupae were found in four tires at densities from 2–958 pupae per liter (see Figure 1), while *Aedes* were sampled in eight tires at densities from 2–52 pupae per L. All tires sampled contained mosquito larvae, including larvae of all species in Table 1. Fourteen tires contained late instar *Ae. albopictus* and thirteen had late instar *Cx. pipiens* larvae. The one tire with 958 *Culex* pupae/L also held 42 *Aedes* pupae/L, and late instar *Cx. pipiens* larvae, *Cx. restuans* larvae and *Ae. albopictus* larvae.

### 4.4. Resident Response and Relative Exposure

A majority of residents in each neighborhood (54–83%) reported being bothered by mosquitoes every day. N4 had both the greatest proportion of residents that reported daily mosquito exposure (83%) and the greatest proportion that reported that they were never bothered by mosquitoes (17%) (Table 3). We calculated a relative risk index of actual mosquito exposure for each neighborhood using data in Table 3. 

Relative Risk Index = Mean # Containers/Neighborhood × Positive Containers (%) × Mean # Pupae/Container

**Table 3:** Neighborhood indicators, calculated across all sampling dates.

NBHD	Median Income	Container #	Mosquito +	Pupae #	Every Day	Never
		Per Parcel		Per Container	Resident Reported Nuisance	
N1	L	1.49	77.0%	14.96	54.0%	8.0%
N2	M	1.71	40.0%	21.00	69.0%	6.0%
N3	L	1.69	73.0%	12.55	54.0%	8.0%
N4	M	2.45	44.0%	2.74	83.0%	17.0%
N5	H	2.11	21.0%	1.32	56.0%	8.0%

**Figure 2:**
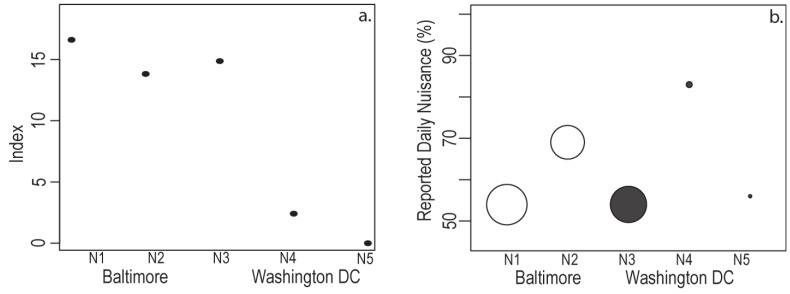
(**a**) The relative index for risk of mosquito exposure is plotted for each neighborhood, shown in order of relative median income in each city. The risk index declines with income within a city and is universally high in the three neighborhoods with median household incomes at or below $40,000 (N1, N2, N3). (**b**) The percentage of residents that reported daily mosquito exposure (y axis) in each Baltimore (white) and Washington (black) neighborhood. Points are scaled by the risk index and larger points indicate greater risk.

This relative index describes broad differences in actual mosquito infestation and likely exposure risk among neighborhoods. The relative risk indices span an order of magnitude from 0.58 in N5 (Washington, high income) to 17.18 in N1 (Baltimore, low income). Our index does not account for differences among container types, nor does it account for differences in human-biting rates among species. Including a habitat importance measure would increase the risk in both Baltimore neighborhoods because tire habitats, the most important predictor of pupae presence and abundance, were only found in Baltimore. Still, risk of mosquito exposure by this index is already greatest in Baltimore and for residents in the lowest median income neighborhoods in both cities (Figure 2(a)). However, our index of risk of exposure to mosquitoes was not predictive of resident-reported nuisance levels within a neighborhood (Figure 2(b)), which were lowest in the two lower income neighborhoods (Table 3). 

## 5. Conclusions

We found that mosquito infestation across neighborhoods in two temperate U.S. cities is heterogeneous and that species composition and abundance vary importantly with economic conditions. Our study contributes to a broader understanding of the interactions and feedbacks between human behavior, perception, and mosquito ecology that are critical drivers of infestation and nuisance patterns in urban settings. Species richness and composition measures differed considerably between Baltimore and Washington (Table 1), despite being in the same ecological region with the same regional species pool. The abundant production of *Cx. pipiens* in Baltimore neighborhoods, predominantly in tire containers, represents a potential increased risk of WNV exposure for Baltimore residents. Tires are widely documented as important habitat for mosquito development [120,121], although little is known about how widespread or productive these habitats are in urban landscapes [79]. It is possible that differences in government mosquito control efforts have resulted in the city-level differences we found, although no neighborhoods were treated during our study. 

Discarded tires were the most important predictor of both *Culex* and *Aedes* pupae production (and implied adult emergence) [122,123] and the abundance of these most productive habitats may vary across neighborhoods in a predictable way. Our findings regarding relative importance of specific anthropogenic container habitats generally agree with both the 2010 DC study [62] and similar work in residential landscapes in New Jersey [79]. Planters were also important habitat for *Aedes* pupae and while these habitats were found in all neighborhoods, 90% of the pupae positive planters were in Baltimore and 50% of those with *Aedes* pupae were found in the lowest income neighborhood. Budget support and community engagement could be streamlined with more information regarding what types of container habitats are most productive and where these containers are most likely to be found. It remains unclear why planters in lower-income neighborhoods were more likely to contain pupae than planters in even the nearby upper income neighborhood and more work is needed to evaluate the importance of resident maintenance *versus* some greater source population in these neighborhoods.

The predictive capacity to identify and effectively manage growing vector populations remains limited at broad and course spatio-temporal scales, although evidence from our study and other published research shows that infestations and risk are heterogeneous at much finer scales [60,61,72,73,76,124,125,126]. This is especially true in urban areas where complex and interacting socio-ecological factors, including vector control attitudes [64], ultimately determine the abundance and composition of mosquito communities and potential pathogen exposure. Mosquito vectors can utilize resident-managed and discarded containers on both public and private property to achieve rapid development and population growth [79,85,120,127] and thus, control measures require resident participation [79,80,127,128,129]. 

We found little association between reported mosquito exposure and our measures of mosquito production within a neighborhood. This warrants further study, especially as mosquito control efforts in many cities are focused on responding to resident complaints or disease incidence. It is possible that larvae and pupae densities are not well correlated with abundance of human-biting adults. Although mosquito populations are thought to be mainly regulated at the larval stage [29], our sampling of larval habitats may not have been a good proxy of human exposure to the biting adult stage. *Culex* and *Aedes* mosquitoes can utilize habitats within the urban landscape besides ground level containers in backyards, including public storm drains and elevated housing gutters. Further work is planned to evaluate the relative importance of these habitats. Nevertheless, managing anthropogenic container habitats is a vital ingredient to integrated mosquito management. Additionally, people’s perceptions of how bothersome mosquitoes are may be shaped both by a range of diverse experiences outside their immediate backyard environment (e.g., via media, past experience) and by their desire to be outdoors in the first place. These gaps in understanding—on the part of researchers, communities, and public health agencies—speak to the need for further examination of the socio-ecological systems in which mosquitoes are embedded. 
